# Dispositional mindfulness and psychological well-being: investigating the mediating role of meaning in life

**DOI:** 10.3389/fpsyg.2024.1500193

**Published:** 2025-01-22

**Authors:** Aamer Aldbyani, Guiyun Wang, Zhang Chuanxia, Yanxia Qi, Jiaojiao Li, Jie Leng

**Affiliations:** ^1^Shandong Xiehe University, Jinan, China; ^2^Thamar University, Dhamar, Yemen; ^3^Renmin University of China, Beijing, China

**Keywords:** hope, life satisfaction, meaning in life, mindfulness, psychological well-being

## Abstract

**Introduction:**

Mindfulness has been widely recognized for its positive impact on various aspects of psychological well-being. Prior research has investigated the role of mindfulness in enhancing hope and life satisfaction as separate constructs. However, limited attention has been given to the simultaneous influence of mindfulness on both hope and life satisfaction and the underlying mechanisms that may explain these relationships. This study examines the role of dispositional mindfulness in fostering hope and life satisfaction concurrently and explores the potential mediating role of meaning in life in this relationship.

**Methods:**

A total of 1,766 Chinese college students from Shandong Xiehe University participated in the study. Participants completed the Mindful Attention Awareness Scale (MAAS) to assess dispositional mindfulness, the Adult Hope Scale (AHS) to measure hope, the Satisfaction with Life Scale (SWLS) to evaluate life satisfaction, and the Meaning in Life Questionnaire (MLQ) to assess their perceived meaning in life. Statistical analyses, including mediation analysis, were conducted to explore the relationships among these variables.

**Results:**

The findings indicated that meaning in life partially mediated the relationship between dispositional mindfulness and both hope and life satisfaction. Specifically, higher levels of dispositional mindfulness were associated with greater hope and life satisfaction, and this relationship was partially explained by increased perceptions of meaning in life.

**Discussion:**

These results underscore the critical role of meaning in life as a mediator in the positive effects of mindfulness on psychological well-being. The findings suggest that interventions aimed at enhancing mindfulness may benefit from incorporating strategies to foster meaning in life, thereby amplifying their impact on hope and life satisfaction. This study advances our understanding of the mechanisms linking mindfulness to key indicators of psychological well-being and highlights the integrative role of meaning in life in these processes.

## Introduction

Mindfulness plays a crucial role in cultivating positive psychological traits that enhance mental well-being. By promoting a balanced approach to life, mindfulness not only influences individuals positively but also has a beneficial impact on those around them. Individuals who practice mindfulness are better equipped to withstand adversity and exercise greater control over their emotions, contributing to overall psychological health. Mindfulness encompasses a variety of strategies designed to improve psychological well-being, life satisfaction, bodily awareness, empathy, and mental clarity (Dobkin and Hassed, 2016). It is often described as a state of consciousness characterized by refined attentional abilities and a nonjudgmental awareness of both internal and external experiences. Research has consistently shown that higher levels of mindfulness are associated with a range of positive outcomes, including enhanced hope and life satisfaction. Programs that integrate mindfulness with hope-based interventions have been developed to foster mindful awareness and encourage these positive psychological attributes (Abdolghaderi et al., 2018; Malinowski and Lim, 2015; Thornton et al., 2014).

Many studies have examined the relationship between mindfulness and various psychological attributes contributing to hope and life satisfaction. However, a more critical and in-depth analysis of these studies is warranted. Research has repeatedly shown that mindfulness is positively linked with several emotional and psychological outcomes, such as increased positive emotions (Du et al., 2019; Garland et al., 2015; Lindsay et al., 2018), emotional regulation (Hill and Updegraff, 2012; Koç and Uzun, 2024; Prakash et al., 2015; Tomlinson et al., 2018), and emotional intelligence (Agastya, 2023; Miao et al., 2018; Pantelides, 2024; Wang, 2023). These findings suggest that mindfulness enhances emotional stability and psychological well-being, both of which are crucial for fostering hope and life satisfaction. However, while the evidence suggests a positive relationship, the specific mechanisms through which mindfulness influences these outcomes remain underexplored. Further research is needed to clarify these underlying processes.

Moreover, mindfulness appears to support individuals in navigating challenging life circumstances by fostering present-moment awareness and self-compassion. These qualities may prevent individuals from experiencing extreme emotional states, thereby contributing to greater psychological well-being. Although the evidence suggests that mindfulness helps individuals cope with stress and emotional difficulties, its impact on life satisfaction and hope may vary depending on individual and contextual factors.

The concept of mindfulness itself deserves further consideration, particularly regarding its role in fostering a goal-oriented mindset. Mindfulness, coupled with a sense of meaning in life, may encourage individuals to take purposeful steps toward a more fulfilling future (Singh and Devender, 2015; Snyder, 2010). However, it is important to critically assess whether mindfulness alone can instill a sense of meaning in life or whether other factors, such as cultural context and personal values, also play significant roles. Existing literature suggests that hope and life satisfaction are influenced by multiple factors, including quality of life (Montgomery et al., 2016; Pais-Ribeiro and Pedro, 2022; Wang et al., 2024), psychological well-being (Siah, 2024; Warsah et al., 2023), and happiness (El Shobaky et al., 2020; Hassan et al., 2018; Kardas et al., 2019; Sevari et al., 2020). These findings imply that mindfulness may be one factor among many influencing these outcomes, but its relative importance requires further investigation.

In addition, emotional intelligence (Delhom et al., 2020; Mousa et al., 2017; Rey et al., 2011; Sarıcam et al., 2015; Sharmin et al., 2019), self-efficacy (D’Souza et al., 2020; Munoz et al., 2017; Zhou and Kam, 2016), and job performance (Duggleby et al., 2009; Kudo et al., 2016; Peterson and Byron, 2008) have been identified as key factors influencing hope and life satisfaction. While mindfulness is likely to play a role in these areas, it is important to examine the interactions between these factors and their collective impact on overall well-being. Furthermore, research has demonstrated that a positive educational environment (Yotsidi et al., 2018), creativity, leadership (Anwar et al., 2019; Rego et al., 2014; Wandeler et al., 2016), and effective stress-coping strategies (Cahapay and Bangoc, 2022; Fruh et al., 2021; Hamid, 2020) are also associated with enhanced hope and life satisfaction. These findings suggest that mindfulness alone cannot fully account for well-being, and other personal and environmental factors must also be considered.

Previous research has laid a foundation for understanding the connections between mindfulness, meaning in life, hope, and life satisfaction. However, the specific role of meaning in life as a mediator in the positive effects of mindfulness on hope and life satisfaction remains unclear. The present study seeks to explore how dispositional mindfulness contributes to enhancing both hope and life satisfaction, with a particular focus on the potential mediating role of meaning in life in this relationship.

### Mindfulness and hope

Mindfulness is a positive mental quality that significantly influences an individual’s mental and psychological development. It enables individuals to lead their lives consciously and independently, fostering emotional awareness that helps them overcome negative feelings while enhancing happiness and satisfaction (Lau et al., 2013). Increased mental attentiveness, which supports better life integration, is a key focus of mindfulness programs designed to raise individuals’ awareness and productivity (Malinowski and Lim, 2015). Mindfulness improves a person’s ability to manage their environment and develop appropriate responses to unpleasant situations by promoting awareness and informed decision-making. It encourages nonjudgmental responses to emotions and feelings (Dobkin and Hassed, 2016; Bluth and Blanton, 2014). Furthermore, mindfulness strengthens a person’s ability to tolerate and regulate negative emotions, contributing to improved emotional resilience (Cho et al., 2017).

Hope is a vital factor that helps individuals accept, cope with, and overcome challenging circumstances. It encourages people to adopt a task-focused approach and take deliberate actions to achieve a better quality of life. Rather than simply improving their own lives, hope fosters constructive and positive interactions and integration within their environments (Snyder, 2002). Hope is linked to several factors crucial for an individual’s success, including professional achievement (Wood, 2022), quality of life (Pais-Ribeiro and Pedro, 2022), psychological well-being (Yalnizca-Yildirim and Cenkseven-Önder, 2023), happiness and optimism (Al-Sadi, 2018; Judeh and Abu-Jarad, 2011), and emotional intelligence (Sharmin et al., 2019). Additional factors associated with hope include self-efficacy (Zhou and Kam, 2016), job performance and satisfaction (Duggleby et al., 2009; Peterson and Byron, 2008), the work and educational environment (Yotsidi et al., 2018), creativity and leadership (Rego et al., 2014), and stress coping strategies (Cahapay and Bangoc, 2022; Fruh et al., 2021; Hamid, 2020).

**Figure 1:**
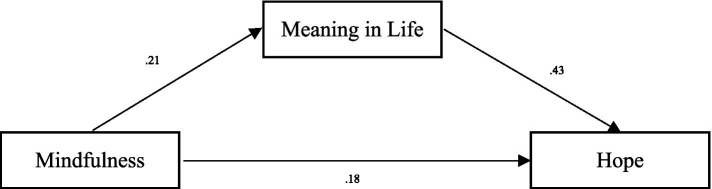
The effect of mindfulness on hope.

According to Snyder et al. (1991), hope is a positive motivational state defined by a sense of accomplishment, goal-directed energy, and readiness to achieve one’s aspirations. Snyder et al. (1996) expanded on this concept by emphasizing its cognitive component, suggesting that hope involves setting meaningful goals, creating actionable plans, and fostering confidence in one’s ability to achieve them. Furthermore, Snyder et al. (1998) highlighted the critical role of hope in empowering individuals to navigate and overcome obstacles and challenges (Figure 1).

Emerging research suggests a strong positive relationship between mindfulness and hope (Heshmati et al., 2024; Malinowski and Lim, 2015; Satici and Satici, 2022; Singh and Devender, 2015). For example, Munoz et al. (2018) found that individuals who practiced mindfulness regularly exhibited higher levels of hope. This connection likely arises from mindfulness’ ability to cultivate present-moment awareness, reduce negative rumination, and enhance self-compassion, all of which contribute to a more hopeful outlook. However, it is important to note that the relationship between mindfulness and hope remains under investigation, and not all studies have found a significant or strong connection (Bhardwaj and Imran, 2023). Further research is needed to fully understand the complex interplay between these two constructs. Based on these findings, our first hypothesis is as follows:

*H1: Dispositional mindfulness positively correlates with hope.*

### Mindfulness and life satisfaction

Mindfulness is a key element that helps develop constructive mental abilities, benefiting a person’s psychological well-being. It enables individuals to lead balanced lives and positively influence others. A mindful person can overcome negativity and maintain full control over their emotions and feelings. Mindfulness encompasses a variety of techniques aimed at enhancing psychological well-being, life satisfaction, physical clarity, empathy, and mental clarity (Dobkin and Hassed, 2016). By practicing mindfulness, individuals learn to accept situations as they are, even when faced with psychological or emotional strain. Rather than avoiding these circumstances, they can confront them directly (Siegel, 2010; Stahl and Goldstein, 2019; Williams and Penman, 2012). This acceptance helps individuals defend against excessive anxiety and promotes awareness of the physiological states associated with their emotions.

The concept of life satisfaction has garnered significant attention in the fields of mental health and adaptability (Diener et al., 2000). Life satisfaction refers to how individuals evaluate their lives from their own perspective across various dimensions, making it a crucial concept for both psychological health and overall well-being (Diener et al., 2013; Pavot and Diener, 2008). This evaluation can be reflected in how individuals perceive their lives as a whole or in how they assess specific areas, such as satisfaction with work, studies, or personal achievements. It can also be influenced by the frequency of positive experiences, which contribute to psychological well-being and happiness, or negative experiences, which can lead to stress, anxiety, and depression, resulting in varying degrees of dissatisfaction (Akokuwebe et al., 2023; Castelli et al., 2023; Roberts et al., 1983).

According to Mace et al. (2024), mindfulness is a fundamental component of sound awareness. It is based on addressing life’s challenges by accepting situations without judgment, ultimately leading to greater life satisfaction. As a result, studies have found a positive relationship between mindfulness and life satisfaction, including research by Chang et al. (2015), Kütük et al. (2023), LeBlanc et al. (2021), Li et al. (2022), Pidgeon and Appleby (2014), Stolarski et al. (2016), and Wang and Kong (2014). A recent study by Zadhasan (2024) examined the roles of locus of control and mindfulness as predictors of life satisfaction. The results indicated that mindfulness significantly predicted life satisfaction, with higher mindfulness scores being associated with greater life satisfaction. This may be due to the strong connection between mindfulness and life satisfaction. Based on these findings, our second hypothesis is as follows:

*H2: Dispositional mindfulness positively correlates with life satisfaction.*

### Meaning in life as a mediator

**Figure 2:**
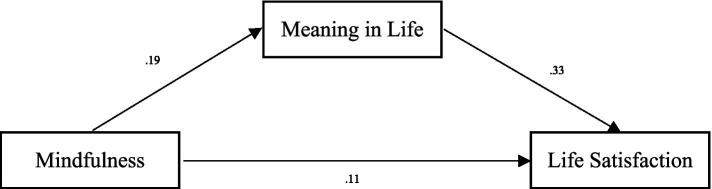
The effect of mindfulness on life satisfaction.

A crucial aspect of psychological well-being is finding purpose in life (Ryff, 1989). It is well-established that having a sense of purpose offers numerous benefits. A sense of purpose has been shown to reduce mortality rates over a lifetime (Hill and Turiano, 2014), improve physical health (Czekierda et al., 2017), and reduce anxiety and the desire for early death in patients with advanced cancer (Breitbart et al., 2010). Mindfulness practices can help individuals find more meaning in life through the development of present-moment awareness, the reduction of negative thoughts, the promotion of self-compassion, and the cultivation of interpersonal connections. These practices, in turn, enhance well-being and foster a more meaningful existence (Allan et al., 2015; Bloch et al., 2017; Chu and Mak, 2020; Hanner, 2024; Koç and Uzun, 2024). Psychological theories elucidate the relationship between mindfulness and meaning in life. For example, Wolf’s framework suggests that mindfulness acts as a mediating factor in the creation of meaning (Hanner, 2024). Chu and Mak (2020) argue that a sense of meaning in life is predicted by positive affect, which results from two tendencies: the inclination to reappraise experiences positively and the disposition to focus on positive experiences. Their meta-analysis revealed a correlation between mindfulness and meaning in life, with the impact of mindfulness being mediated by decentering, authentic self-awareness, and attention to positive experiences (Figure 2).

On the other hand, numerous studies have shown that individuals who find meaning in their lives tend to report higher life satisfaction and view the future more optimistically, which enhances their level of hope (Dursun, 2012; Krok, 2018; Peterson, 2000; Santos et al., 2012). A study by Karataş et al. (2021) examined the relationships between meaning in life, hope, life satisfaction, and COVID-19-related fear. The results revealed positive correlations between meaning in life, hope, and life satisfaction, with meaning in life serving as a strong predictor of life satisfaction. Similarly, a study by Bhardwaj and Imran (2023) found a positive relationship between meaning in life, hope, and life satisfaction.

While previous studies have laid the groundwork for understanding the relationship between meaning in life, hope, and life satisfaction, the specific role of meaning in life as a mediator of the positive effects of dispositional mindfulness on hope and life satisfaction remains unclear. Our study is among the first to examine the role of meaning in life as a mediator between dispositional mindfulness, hope, and life satisfaction. This hypothesis is supported by mindfulness theory in the existing literature (Elices et al., 2019; Follette and Hazlett-Stevens, 2016; Langer and Moldoveanu, 2000; Schonert-Reichl and Roeser, 2016).

Meaning in life theory suggests that having a sense of meaning is closely related to psychological well-being, as individuals who find meaning tend to experience higher levels of hope and life satisfaction (Eagleton, 2007). According to a behavioral model, setting goals that align with personal values enhances the sense of meaning (Klein, 2017; Schippers and Ziegler, 2019). Hope, as described in hope theory, is a fundamental psychological factor that influences behavior and well-being. Research indicates a positive correlation between hope and meaning in life, with hopeful individuals viewing their futures more optimistically (Dursun, 2012; Hedayati and Khazaei, 2014; Peterson, 2000; Yalçın and Malkoç, 2015). Mindfulness enhances self-awareness, reduces negative thoughts, and fosters self-compassion (Evans et al., 2018; Neff and Tirch, 2013; Raab, 2014; Viskovich and De George-Walker, 2019). When individuals perceive their lives as meaningful, their sense of hope increases, motivating them to set and pursue goals, which in turn elevates their levels of hope and life satisfaction. Based on these findings, our third hypothesis is as follows:

*H3: Meaning in life may mediate the relationship between dispositional mindfulness, hope, and life satisfaction.*

## Method

This study employed a quantitative, cross-sectional design to investigate the relationships between dispositional mindfulness, meaning in life, hope, and life satisfaction among Chinese university students. Specifically, we examined the mediating role of meaning in life in the association between dispositional mindfulness and both hope and life satisfaction. Furthuremore, the cross-sectional design was chosen for its efficiency in exploring relationships between variables at a single point in time. This approach is well-suited for examining the associations between mindfulness, meaning in life, hope, and life satisfaction in a large sample, thereby identifying potential patterns that warrant further investigation. Although a longitudinal design would provide stronger evidence for causality, our cross-sectional approach serves as a necessary first step in elucidating the complex relationships between these factors in the specific population of Chinese university students.

### Participants

A total of 1,766 college students from Shandong Xiehe University in Jinan, China, voluntarily participated in this study. They provided informed consent, ensuring the privacy of their responses. The age of the participants ranged from 18 to 22 years (*M* = 20.08, SD = 2.44). None of the participants had any prior experience with mindfulness training or related disciplines, such as Tai Chi, Qigong, or yoga. Table 1 presents the demographics of the study participants.

**Table 1:** Demographics of the study participants.

Variable	Frequency	Percent%
Gender		
Male	698	39.52%
Female	1,068	60.48%
Grade		
1st	69	3.91%
2nd	1,008	57.08%
3rd	613	34.71%
4th	76	4.3%
Family income		
3,000–10,000	1,336	75.65%
10,000–20,000	339	19.2%
20,000-above	91	5.15%
Total	1766	100%

This study focused on Chinese university students for several key reasons. First, the transition from adolescence to young adulthood is a critical developmental stage, characterized by increased academic pressure, social adjustments, and the formation of personal identity and values. These factors can significantly impact mental well-being and the development of positive psychological constructs, such as hope and life satisfaction. Second, cultural values in China, which emphasize collectivism and academic success, may influence how mindfulness, meaning in life, hope, and life satisfaction are experienced. Studying this population allows us to explore these relationships within a specific cultural and developmental context, potentially contributing valuable insights into mental health interventions and educational programs tailored to this context. Additionally, university students often face stressors that could benefit from mindfulness and related practices, making them a relevant population for exploring these connections. Finally, using a student sample provides a readily accessible group for conducting this type of study. However, the specificity of the findings from this student sample must be acknowledged, as they may not directly generalize to other populations (e.g., adults outside of university or individuals in other countries). Further research in other populations is needed to establish the generalizability of the findings.

### Procedures

Data were collected between August and September 2024 using online questionnaires administered during regularly scheduled classes, after securing the necessary permissions from university officials and relevant academic departments. Participants were given approximately 45–60 min to complete all measures. Research assistants were present to answer questions and oversee the administration of the questionnaires, ensuring standardized procedures. The questionnaires were organized in a specific order.

The study procedures were approved by the Ethics Committee of Shandong Xiehe University. All procedures were conducted in accordance with the ethical principles outlined in the Declaration of Helsinki. Informed consent was obtained from all participants prior to their participation.

### Instruments

#### Mindfulness

The Chinese version of the Mindful Attention Awareness Scale (MAAS), which contains 15 items (Brown and Ryan, 2003), has been proven to be a reliable and valid scale in Chinese settings (e.g., Chen et al., 2012). Each item was rated on a 6-point Likert scale, with one indicating “*Almost always*” and six indicating “*Almost never*.” In this study, Cronbach’s alpha was *0.88*.

#### Hope

The Chinese version of the Adult Hope Scale (AHS), which contains 12 items (Snyder, 1995), has been proven to be a reliable and valid scale in Chinese settings (e.g., Sun et al., 2012). Each item was rated on a 4-point Likert scale, ranging from one (*Definitely false*) to four (*Definitely true*). In this study, Cronbach’s alpha was 0.91.

#### Life satisfaction

The Chinese version of the Satisfaction with Life Scale (SWLS), which contains 5 items (Diener et al., 1985), has been proven to be a reliable and valid scale in Chinese settings (e.g., Xiong and Xu, 2009). Each item was rated on a 7-point Likert scale, ranging from one (*Strongly disagree*) to seven (*Strongly agree*). In this study, Cronbach’s alpha was 0.89.

#### Meaning in life

The Chinese version of the Meaning of Life Questionnaire (MLQ), which contains 10 items (Steger et al., 2006), has been proven to be a reliable and valid scale in Chinese settings (e.g., Liu and Gan, 2010). Each item was rated on a 7-point Likert scale, ranging from one (*Absolutely untrue*) to seven (*Absolutely true*). In this study, Cronbach’s alpha was 0.90.

### Data analysis

Pearson’s correlation coefficients were calculated to examine the relationships between the study variables. Mediation analyses were conducted using the PROCESS macro (version 3.5) in SPSS. To estimate the 95% confidence intervals for the mediated effects in the models, 5,000 bootstrap resamples were generated (Hayes, 2012).

## Results

### Correlation among study variables

The results (Table 2) show that all the study variables are positively correlated with each other.

**Table 2:** Correlation among the study variables.

Variables	1	2	3	4
1. Mindfulness	1			
2. Hope	0.423	1		
3. Life satisfaction	0.498	0.547	1	
4. Meaning in life	0.477	0.551	0.563	1

*p < 0.05.

### Mediation effects

To investigate the influence of mindfulness on hope, the bias-corrected percentile bootstrap method (Model 4 in SPSS Macro PROCESS, sample = 5,000, 95% CI) was used to test the mediating effect. When mindfulness was the independent variable, hope and life satisfaction were the dependent variables, and meaning in life was the mediating variable, the results were as follows:

### Hope

After controlling for factors that might influence both mindfulness and hope, such as gender, age, grade, experience, and monthly family income, all control variables’ effects were non-significant. The total effect of mindfulness was significant (*β* = 0.27, *p* < 0.05), and the direct effect of mindfulness on hope was also significant (*β* = 0.18, *p* < 0.05), indicating that mindfulness positively predicted hope. Additionally, meaning in life positively predicted hope. The bias-corrected percentile bootstrap method showed that the indirect effect of mindfulness on hope through meaning in life was significant, ab = 0.09, SE = 0.02, 95% CI = [0.05, 0.13]. These results suggest that meaning in life partially mediated the effect of mindfulness on hope. For more details, see Table 3.

**Table 3:** The effect of mindfulness on hope.

Model	*b*	SE	95% CI
Mindfulness → Meaning in life (a)	0.21	0.02	[0.17, 0.25]
Meaning in Life → Hope (b)	0.43	0.04	[0.35, 0.51]
Mindfulness → Hope (c’)	0.18	0.02	[0.14, 0.22]
Mindfulness → Meaning in life → Hope (ab)	0.09	0.02	[0.05, 0.13]
Total effect of mindfulness→ Hope (c)	0.27	0.02	[0.23, 0.31]

### Life satisfaction

After controlling for factors that might influence both mindfulness and life satisfaction, such as gender, age, grade, experience, and monthly family income, all control variables’ effects were non-significant. The total effect of mindfulness was significant (*β* = 0.17, *p* < 0.05), and the direct effect of mindfulness on life satisfaction was significant (*β* = 0.11, *p* < 0.05), indicating that mindfulness positively predicted life satisfaction. Additionally, meaning in life positively predicted life satisfaction. The bias-corrected percentile bootstrap method showed that the indirect effect of mindfulness on life satisfaction through meaning in life was significant, ab = 0.06, SE = 0.02, 95% CI = [0.05, 0.12]. These results suggest that meaning in life partially mediated the effect of mindfulness on life satisfaction. For more details, see Table 4.

**Table 4:** The effect of mindfulness on life satisfaction.

Model	*b*	SE	95% CI
Mindfulness → Meaning in life (a)	0.19	0.02	[0.15, 0.22]
Meaning in Life → Life satisfaction (b)	0.33	0.04	[0.25, 0.84]
Mindfulness → Life satisfaction (c’)	0.11	0.02	[0.07, 0.14]
Mindfulness → Meaning in life → Life satisfaction (ab)	0.06	0.02	[0.05, 0.12]
Total effect of mindfulness → Life satisfaction (c)	0.17	0.02	[0.17, 0.24]

## Discussion

The first aim of this study was to investigate the relationship between dispositional mindfulness, meaning in life, hope, and life satisfaction. The results of our study revealed that dispositional mindfulness is positively correlated with meaning in life, hope, and life satisfaction. These findings are partially consistent with several studies that have also found positive correlations between mindfulness and meaning in life (Allan et al., 2015; Chu and Mak, 2020; Li et al., 2022; Tan et al., 2021), mindfulness and hope (Malinowski and Lim, 2015; Satici and Satici, 2022; Singh and Devender, 2015), and mindfulness and life satisfaction (Abbasi et al., 2024; Bajaj and Pande, 2016; Kan et al., 2024; Li et al., 2022; Stolarski et al., 2016). These results highlight mindfulness’s role in enhancing present-moment awareness, engaging fully in current experiences, adopting a nonjudgmental perspective, and identifying what is important, all of which foster self-compassion and interpersonal connections—key elements for deriving meaning in life. Furthermore, individuals with higher dispositional mindfulness are more likely to re-evaluate situations positively and focus on positive experiences, which in turn enhances their sense of hope and life satisfaction.

The second aim of our study was to explore the possibility that meaning in life mediates the relationship between dispositional mindfulness, hope, and life satisfaction. The results confirmed our hypothesis that meaning in life partially mediated this relationship. Although this study is the first to examine this role, previous theoretical literature supports this finding through mindfulness theory, meaning in life theory, and hope theory.

Mindfulness theory emphasizes the importance of present-moment awareness and acceptance in enhancing mental health, helping individuals better understand their thoughts and feelings (Baer and Huss, 2008; Elices et al., 2019; Follette and Hazlett-Stevens, 2016). This understanding can lead to deeper experiences of meaning in life. In parallel, meaning in life theory suggests that having a sense of meaning is closely linked to psychological well-being (Eagleton, 2007). Individuals who find meaning in their lives tend to report higher levels of hope and satisfaction (Cotton Bronk et al., 2009). According to a behavioral model, when individuals set goals that align with their personal values, their sense of meaning is enhanced (Klein, 2017; Schippers and Ziegler, 2019).

Hope theory further posits that hope is an essential psychological factor influencing behavior and well-being. Research indicates that hope is positively correlated with meaning in life, as hopeful individuals tend to view their futures more optimistically (Bailey et al., 2007; Dursun, 2012; Peterson, 2000). Mindfulness promotes self-awareness, helping individuals recognize their values and goals. By reducing negative thoughts and fostering self-compassion, mindfulness can lead to an increased sense of meaning in life. When individuals perceive their lives as meaningful, it enhances their sense of hope (Bloch et al., 2017; Scheier and Carver, 1993). Meaning in life motivates individuals to set and pursue their goals, thus increasing hope and life satisfaction, as individuals who are satisfied with their lives find value in their experiences.

Several limitations should be considered when interpreting the findings of this study. One major limitation is the use of self-report questionnaires, which may introduce bias into the data. For instance, social desirability bias may lead individuals to overreport socially favorable behaviors or underreport undesirable ones, potentially inflating the correlations observed in this study. Therefore, further research using objective measures is needed to confirm these results.

Another limitation is that our sample was drawn from undergraduate students at a single university in China, which limits the generalizability of our findings. While we provided a rationale for using this sample in the methods section, it is important to recognize that the observed associations may be specific to this group. Caution should be exercised when applying these conclusions to other populations. For example, our sample consisted of university students within a specific age range and without prior mindfulness experience, further limiting generalizability. Differences in cultural context, age, or educational background may influence the relationships between mindfulness, meaning in life, hope, and life satisfaction.

Additionally, the cross-sectional design of this study prevents us from drawing causal inferences about the relationships between the variables. While we observed significant associations and established a mediating role, we cannot conclude whether increased mindfulness causes an increase in meaning in life, for example. Longitudinal studies are needed to establish causality.

Finally, we did not account for potential variables that might affect the results, such as academic stress, mental health conditions, and socioeconomic status. These factors may also influence the relationships examined in this study. Future research should consider these variables to provide a more comprehensive understanding of the dynamics between mindfulness, meaning in life, hope, and life satisfaction.

Future research should expand sample diversity by replicating this study across diverse samples encompassing various age groups, ethnic backgrounds, socioeconomic levels, and educational institutions to evaluate the generalizability of our findings. Furthermore, longitudinal designs should be used to establish the directionality of the observed relationships. This will allow us to better understand cause and effect and how mindfulness, meaning, hope, and life satisfaction change over time. Researchers should also employ multiple measurement methods, supplementing self-report measures with other methods, such as behavioral assessments and neurobiological measures, to reduce potential bias. In addition, it will be important to investigate other potential mediators and moderators influencing the connections between mindfulness, meaning in life, hope, and life satisfaction. This includes examining factors like academic stress, mental health conditions, and cultural values. Finally, intervention studies should be conducted where mindfulness-based programs are implemented to directly examine the effects of increasing mindfulness on meaning in life, hope, and life satisfaction and future studies should explore the empirical connections between other forms of mindfulness practice (e.g., meditation, movement-based practices) and how they relate to other aspects of student life, such as personality and academic performance.

## Conclusion

This study highlights the positive relationships between dispositional mindfulness, meaning in life, hope, and life satisfaction. Our findings demonstrate that mindfulness not only enhances individuals’ present-moment awareness and self-compassion but also fosters a deeper sense of meaning, which, in turn, boosts their hope and overall life satisfaction. Additionally, the results confirm the mediating role of meaning in life in the relationship between mindfulness and both hope and life satisfaction, suggesting that mindfulness plays a critical role in shaping psychological well-being through its impact on meaning. However, the study’s reliance on self-report measures presents limitations, particularly concerning potential biases toward socially desirable responses. Future research should address these limitations by incorporating more diverse populations and exploring alternative methods of data collection. Expanding the scope of research will help determine whether these findings are generalizable across different educational contexts. Moreover, exploring the relationship between mindfulness and other aspects of student life, such as personality and academic performance, could provide further valuable insights. Ultimately, this study underscores the importance of mindfulness in enhancing well-being and offers a foundation for further exploration in both academic and personal contexts.

## Data Availability

The raw data supporting the conclusions of this article will be made available by the authors, without undue reservation.
